# A Robust Technique for Polymer Damping Identification Using Experimental Transmissibility Data

**DOI:** 10.3390/polym14132535

**Published:** 2022-06-21

**Authors:** Mikel Brun, Fernando Cortés, Jon García-Barruetabeña, Imanol Sarría, María Jesús Elejabarrieta

**Affiliations:** Department of Mechanics, Design and Industrial Management, University of Deusto, Avda. de las Universidades 24, 48007 Bilbao, Spain; fernando.cortes@deusto.es (F.C.); jgarcia.barruetabena@deusto.es (J.G.-B.); isarria@deusto.es (I.S.); maria.elejabarrieta@deusto.es (M.J.E.)

**Keywords:** characterisation, polymers, viscoelasticity, high damping, curve fitting

## Abstract

This paper presents a robust method to estimate polymers’ damping, based on modal identification methods on frequency functions. The proposed method presents great advantages compared to other traditional methods such as the HPB method for polymeric materials where high damping or noise levels can limit their use. Specifically, this new method is applied on an experimental transmissibility function measured in a composite cantilever beam and the complex modulus is determined as a function of frequency. From this, a regenerated function is obtained based on the Euler–Bernoulli beam theory, and it is compared with experimental data. It can be concluded that the best way to apply the curve-fitting method for further testing of polymeric materials is when it is used with the whole frequency range by means of the MDOF method considering the residuals. In addition, this has the added advantage that the number of experimental tests to be carried out is much lower compared to using the SDOF method.

## 1. Introduction

Polymeric materials are widely used in engineering applications and many studies on their mechanical behaviour are available [1,2,3]. The characterisation of polymeric materials is a recurrent topic of research, and many contributions are being developed continuously [4,5,6]. Determining the damping of a polymeric material is of special importance for applications aimed at reducing vibrations.

The mechanical properties characterisation of polymeric materials requires devoted experimental techniques, and identifying the correct one is of paramount importance, especially in engineering applications involving high-damping materials. In effect, among the dynamic properties of polymers, the damping is of great interest due to the fact that it is the most difficult property to determine and the most sensitive one to external factors. There are known techniques for characterising materials in the time domain as well as in the frequency domain. Generally, for low-damping levels, time-domain techniques provide better results than frequency-domain techniques, whereas for high-damping levels, the opposite is true [7,8]. Frequency methods consist in measuring a frequency response and in determining mainly the resonance frequencies and the damping ratio. However, this can be a challenging task because damping is a very sensitive parameter and its determination highly depends on the experimental data quality, which in some cases can be hard to get.

Polymers have a frequency-dependent complex modulus and thus characterisation techniques in the frequency domain are of special interest. Most of the frequency domain techniques are based on the extraction of the modal parameters from a frequency response, mainly the resonance frequency and the modal loss factor. At the same time, modal parameters extraction methods can be classified with single-degree-of-freedom methods (SDOF) and multiple-degrees-of-freedom methods (MDOF), the main difference between them being whether a single mode or multiple modes are considered at once in the identification of the modal parameters.

Among SDOF techniques, one of the traditional methods used to identify the properties of polymers in the frequency domain is the half-power bandwidth (HPB) method due to the simplicity of its implementation [9]. This method consists in identifying the resonance frequencies at the maximum of the peaks of a frequency domain function and calculating the loss factor by relating the bandwidth of the resonance peaks with their corresponding resonance frequencies. The HPB method is widely spread in many fields and can be found in many applications: to characterise a polymer matrix composite with different angles of fibre orientations [10], to determine the properties of basalt fibre-reinforced composites with nanoclay particles [11], to determine the damping of polymeric beams with a pyramidal truss core [12] and to determine the storage modulus of various systems [13]. For the specific case of viscoelastic materials, the HPB method is the default method recommended by the standard ASTM E756-05 [14].

**Figure 1:**
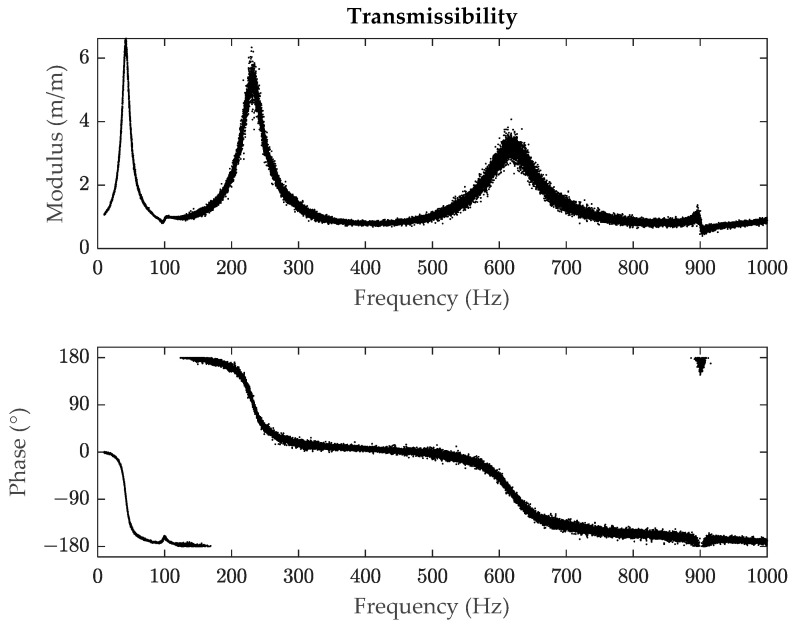
Example of a transmissibility signal (modulus and phase) with noise of a polymeric material.

**Figure 2:**
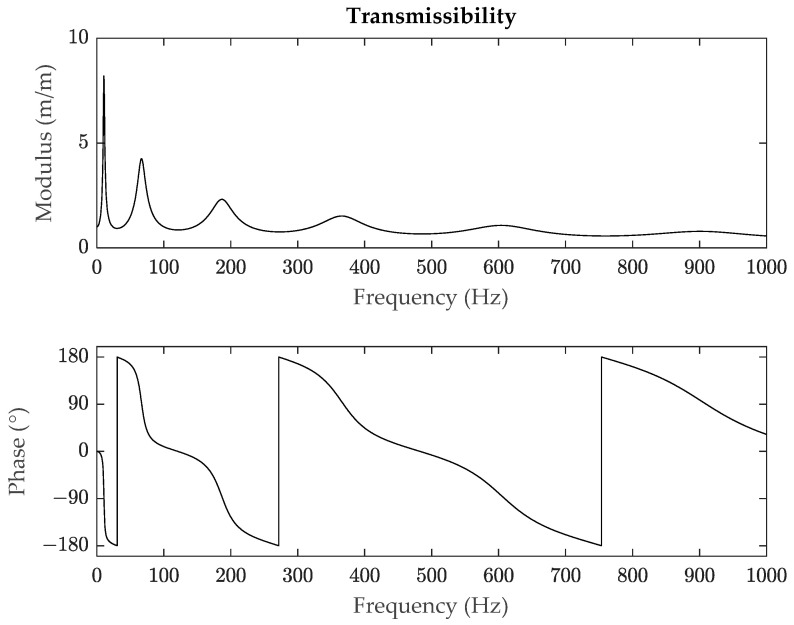
Example of a transmissibility signal (modulus and phase) of a polymeric material with high damping.

However, despite its common use, the HPB method has limitations. When a system presents a high damping or the experimental data in the frequency domain are not accurate enough due to noise, which can be common in practical testing, this method does not provide solid results [15], mainly based on the fact that when calculating the loss factor, only three points are considered, and the accuracy is low when there is noise. This is shown in Figure 1 (unpublished work) where the second and third modes have noise, in a case in which it is difficult and time consuming to get clean measures. Additionally, the HPB method can be unable to identify the bandwidth of a resonance peak if the damping is high as it can be seen in Figure 2 (unpublished work), where for the sixth mode, the amplitude of the resonance peak is not reduced by 3.01 dB. Studies on the error estimation of this method have been performed for example in [16], and ultimately analyses have also been made to implement algorithms to reduce this error such as in [15].

Among MDOF techniques, the curve-fitting method is common where an analytical expression is fitted to experimental data [9] in order to extract the dynamic properties. The analytical expression used in this method usually has some terms called residuals that consider the effect that the low and high modes have on the frequency response, given that usually, not all existing modes can be measured at the same time. Additionally, this method can be used as a SDOF technique if only one single mode is measured and used in the analytical expression when fitting to experimental data. There are also other MDOF techniques such as the rational fraction polynomial method (RFPM) [17]. Further information for comparisons between different characterisation techniques applied to different structures can be found in [18,19,20,21]. However, most of the studies where these techniques are used are focused on determining the modal properties of structures and not the mechanical properties of a given material. For this reason, in this paper the curve-fitting method is studied to identify the dynamic properties of a polymer from the experimental transmissibility function, with the idea of using this method to accurately characterise high-damping materials such as polymers. Therefore, a method valid for high-damping materials and that can mitigate noise effects is desired here.

Furthermore, this paper aims to contribute to the characterisation of polymeric materials by using the curve-fitting method to identify the dynamic properties of materials, specifically damping, which is the most sensitive and difficult parameter to determine. First, the experimental setup to obtain the transmissibility function of a cantilever viscoelastic beam is described. Next, the numerical procedure to extract the modal parameters and consequently the mechanical properties of the material is developed. Then, an in-depth study is performed with the curve-fitting method used in four different cases, which consist in considering one or multiple resonances with and without residuals, and the corresponding mechanical properties are obtained. Finally, from the obtained mechanical properties, a validation of the results is done by regenerating the original signal according to the Euler–Bernoulli beam theory [22]. Results indicate that the proposed curve-fitting method can regenerate the original signal whereas the HPB method cannot be applied due to the existing noise and high damping of the experimental data.

## 2. Experimental Setup and Data

In this Section, the experimental techniques are presented. First, the specimen is described and next, the apparatus and the measurement chain. Finally, the measured transmissibility function, with which the study is carried out, is presented.

### 2.1. Specimen

A viscoelastic composite beam was tested. It consisted of two thin constraining metallic layers and a polymeric core. The specimen was symmetrical, meaning that the materials and the thicknesses of the constraining layers were equal. The geometry and properties of the specimen are shown in Table 1, where $L_{\mathrm{tot}}$ is the total length of the beam, b is the width of the beam, *H* is the total thickness of the beam, *m* is mass and *L* is the free length of the beam.

The metallic layers were made of galvanised steel DX51 Z275. The polymeric core had a polyester base due to its good adhesion to metals. According to the supplier specifications, this was an amorphous polyester adhesive of medium molecular weight (10,000 kg/kmol) and low glass transition temperature (*T*_g_ = −5 °C). This polymer can be used for thicknesses ranging from 20 to 50 µm. Specifically, the polymeric layer of this specimen had a thickness of 35 µm. The solid content was 45% in mass and 55% of the solvent content was composed of 7.5% butyl acetate, 14.5% xylene, 31.0% methoxypropylacetate-2 and 2.0% isobutanol. It was cured at 200 °C for 60 s. The resulting density of the core layer was 1140 kg/m^3^.

### 2.2. Experimental Setup

**Figure 3:**
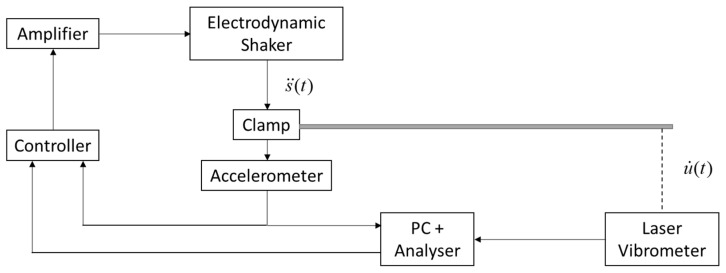
Experimental configuration diagram.

The experimental procedure was based on the standard ASTM E756-05 that states that an excitation force is applied to a cantilever sample employing noncontacting transducers to obtain a frequency response function. To avoid using force transducers, in this work, a base motion was induced to the cantilever-beam-like sample to obtain the transmissibility signal at the measured point instead of the frequency response function, as it was proposed in [23]. The measured point was established at 5 mm from the free end of the cantilever beam. The experimental configuration is illustrated in Figure 3.

**Figure 4:**
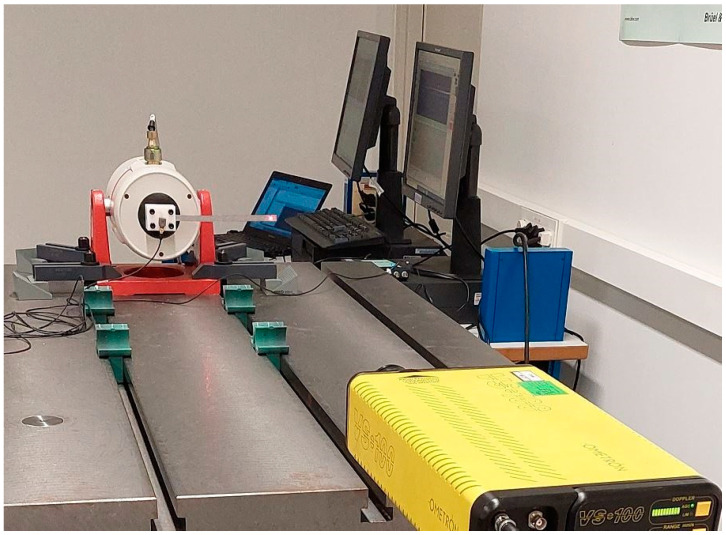
Experimental setup.

The applied base motion acceleration $\ddot{s}(t)$ was a white noise in the frequency range of 0 to 1 kHz generated by an electrodynamic shaker LDS 406. It was measured by an accelerometer ICP Brüel & Kjær 4321 while being loopback-controlled by a vibration controller LASER USB 2.0 with 4 channels. The velocity at the measuring point of the cantilever beam $\dot{u}(t)$ was measured by means of a laser vibrometer Ometron VS 100. Data acquisitions were done on a PC with Photon software in real time. Signal processing in the frequency domain was performed with BK Connect software, where the time signals were transformed to the frequency domain and the transmissibility function was obtained. The data acquisition time was 120 s for a total of 51,600 data points, meaning that a resolution of 0.019 Hz was reached for the measurement. All data were acquired at room temperature. Figure 4 shows an image of the experimental setup.

The transmissibility function was obtained by relating the acceleration at the free end of the beam and the acceleration at the base, and it was defined as

(1) $$T^{*}\left(f\right)=\frac{U^{*}\left(f\right)}{S^{*}\left(f\right)}$$

where f is the frequency, $U^{*}\left(f\right)$ is the Fourier transform of the displacement at the designated point of the cantilever beam u(t) and $S^{*}\left(f\right)$ is that of the displacement at the base s(t), which are obtained from the derivatives of their corresponding signals. The asterisk superscript index ${\left(•\right)}^{*}$ signifies that the magnitude is complex. It was verified that the vibration of the specimen was lineal and that the signal was invariant.

### 2.3. Experimental Data

**Figure 5:**
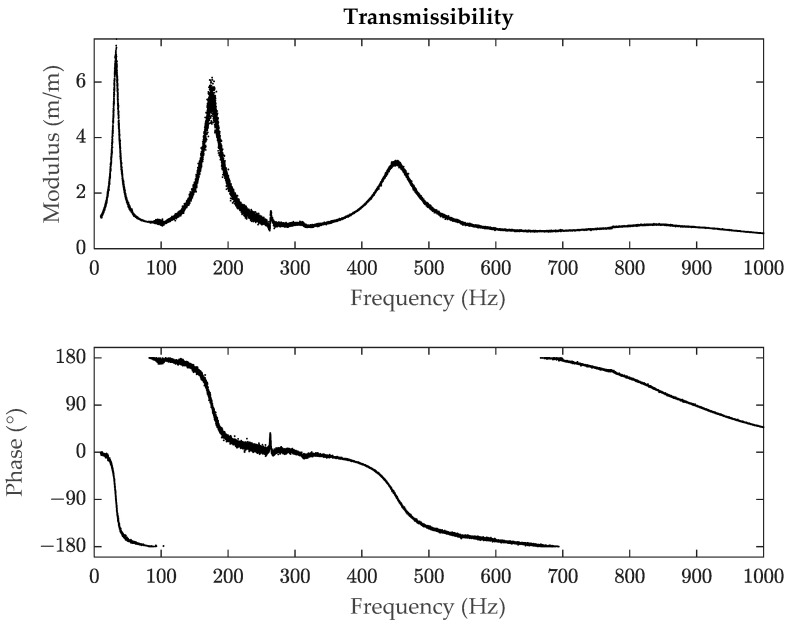
Experimental transmissibility data (modulus and phase) for the tested specimen.

The measured transmissibility function for the composite specimen is illustrated in Figure 5.

According to the experimental data shown in Figure 5, the transmissibility signal presents noise, especially for the second resonance. Additionally, the peak of the fourth resonance has a much lower value compared to the other peaks, implying that the damping of that resonance is high. High levels of noise imply that the HPB method is not applicable to the polymeric specimen due to the experimental data dispersion. At the same time, the HPB method cannot be applied to all the resonances because the low value at the peak of the fourth one prevents it. This is caused because the bandwidth cannot be established since no values are found when the peak is reduced by 3.01 dB. Noisy data can occur when testing viscoelastic polymeric materials, and the nature of a polymeric material can make the HPB method inapplicable when the damping is high, and therefore methods that can mitigate those errors are in general necessary.

## 3. Numerical Procedure

In frequency-domain testing, the main goal is to obtain the frequency-dependent complex modulus of the material, mainly the storage modulus and the loss factor. For that purpose, the resonance peaks of the transmissibility function obtained from raw experimental data were analysed in this work for the previously described cantilever beam configuration.

The resonance frequency and the loss factor at each resonance peak were obtained by means of curve-fitting methods. Specifically, SDOF methods considered a singular resonance while an MDOF method considered multiple resonances of the transmissibility function simultaneously. In both cases, the effect that the residuals had on results was analysed as well. Finally, from the identified resonance frequency, the storage modulus was determined at that frequency.

Although the MDOF method considering the residuals was the most complete case to use in the curve-fitting method, an exhaustive study was performed with these four cases to analyse the robustness of the method. Due to the iterative nature of the curve-fitting method, the SDOF method without residuals was interesting to analyse as well in order to use it to establish valid initial conditions before applying the MDOF method.

### 3.1. Curve-Fitting Method

An analytical expression was fitted to the raw data gathered for the transmissibility function to identify the resonance frequencies and to obtain their modal loss factor. The analytic equation of the transmissibility function was derived from the expression given by [9], from where the transmissibility function was calculated as

(2) $$T^{*}\left(f\right)=\sum_{s=m_{1}}^{m_{n}}\frac{A_{s}^{*}\left(1+i\eta_{s}\right)}{1-{\left(\frac{f}{f_{s}}\right)}^{2}+i\eta_{s}}+\frac{1}{K^{*}}-\frac{1}{{\left(2\pi f\right)}^{2}M^{*}}$$

where $A_{s}^{*}$ is a parameter of the *s*th resonance, $\eta_{s}$ is the modal loss factor at the *s*th resonance, $m_{1}$ is the first analysed resonance, $m_{n}$ is the *n*th analysed resonance, $K^{*}$ is the normalised stiffness residue, $M^{*}$ is the normalised mass residue and $i=\sqrt{-1}$. The residuals considered the effect of the lower and higher resonances outside the analysed frequency range. The parameter extraction of the terms in Equation (2) was carried out by minimising the next objective function g(x),

(3) $$g(x)=\sum_{k=1}^{k_{\max}}\left|T_{\exp,k}^{*}-T^{*}\left(f_{k}\right)\right|$$

where k refers to each applied excitation frequency, $T_{\exp}^{*}$ is the experimental data at each excitation frequency and $T^{*}\left(f_{k}\right)$ is the analytic transmissibility function given by (2) at each excitation frequency. The unknown vector x of the objective function (3) is composed of the parameters $A_{s}^{*}$, $f_{s}$, $\eta_{s}$, for each resonance and $K^{*}$ and $M^{*}$ according to the transmissibility function given by Equation (2) from the experimental data acquired. To solve x, minimisation algorithms have to be used, the Nelder–Mead minimisation algorithm [24] being the one implemented in the *fminsearch* function of MATLAB. This is an iterative method which means that a guess or initial value must be provided for each unknown parameter. This implies that a good approximation of the guess value will improve convergence and accuracy.

### 3.2. Storage Modulus

The numerical procedure used to calculate the storage modulus *E* was based on the Euler–Bernoulli beam theory which is valid if the cross-section dimensions are much lower than the length of the beam. Following this theory, the resulting storage modulus was

(4) $$E\left(f_{s}\right)=\frac{12\rho L^{4}f_{s}^{2}}{H^{2}C_{s}^{2}}$$

where $C_{s}$ is the coefficient of the *s*th mode for a clamped-free beam configuration as shown in the standard ASTM E756-05, $f_{s}$ is the frequency at the *s*th mode, and ρ is the homogenised density which is calculated as

(5) $$\rho =\frac{m}{L_{\mathrm{tot}}bH}$$

according to the terms on Table 1.

### 3.3. Regenerated Signals

To compare the performance of the four methods analysed, regenerated signals from the obtained mechanical properties were compared with the experimental data. The regenerated signals were obtained from the analytic response of a cantilever beam, and it is deduced next.

According to the Euler–Bernoulli beam theory [22], the dynamic beam equation for the tested specimen where no external force was applied was given by

(6) $$EI\frac{\partial^{4}v(x,t)}{\partial x^{4}}+\rho S\frac{\partial^{2}v(x,t)}{\partial t^{2}}=0$$

where x is the spatial variable, *t* is time, *S* is the cross-sectional area and *I* represents the cross-sectional second-order moment (also known as the moment of inertia of the cross-section). By separating the variables in the transverse displacement field by means of a space-dependent term V(x) and a time-dependent term q(t), meaning that v(x,t)=V(x)q(t), and by assuming a harmonic unitary response, Equation (6) can be expressed as

(7) $$\frac{d^{4}V(x)}{dx^{4}}-\lambda^{*}{\left(f\right)}^{4}V(x)=0$$

where the complex eigenvalue $\lambda^{*}$ depends on the frequency, and it is defined as

(8) $$\lambda^{*}\left(f\right)=\sqrt[{4}]{\frac{{\left(2\pi f\right)}^{2}\rho S}{B^{*}\left(f\right)}}$$

where $B^{*}$ is the flexural stiffness given by

(9) $$B^{*}\left(f\right)=E^{*}\left(f\right)I$$

where the frequency dependent complex modulus $E^{*}\left(f\right)$ is

(10) $$E^{*}\left(f\right)=E\left(f\right)\left(1+i\eta \left(f\right)\right)$$

where E(f) is the storage modulus and η(f) is the loss factor. The general solution of Equation (7) takes the form

(11) $$T^{*}\left(f,x\right)=A^{*}\cos\left(\lambda^{*}x\right)+B^{*}\sin\left(\lambda^{*}x\right)+C^{*}\cosh\left(\lambda^{*}x\right)+D^{*}\sinh\left(\lambda^{*}x\right)$$

where $A^{*}$, $B^{*}$, $C^{*}$ and $D^{*}$ are dependent on the boundary conditions. For cantilever beams, those are given by

(12) $$A^{*}=\frac{1+\cos(\lambda^{*}L)\cosh(\lambda^{*}L)-\sin(\lambda^{*}L)\sinh(\lambda^{*}L)}{2\left(1+\cos(\lambda^{*}L)\cosh(\lambda^{*}L)\right)}$$

(13) $$B^{*}=\frac{\cos(\lambda^{*}L)\sinh(\lambda^{*}L)+\sin(\lambda^{*}L)\cosh(\lambda^{*}L)}{2\left(1+\cos(\lambda^{*}L)\cosh(\lambda^{*}L)\right)}$$

(14) $$C^{*}=\frac{1+\cos(\lambda^{*}L)\cosh(\lambda^{*}L)+\sin(\lambda^{*}L)\sinh(\lambda^{*}L)}{2\left(1+\cos(\lambda^{*}L)\cosh(\lambda^{*}L)\right)}$$

and

(15) $$D^{*}=-\frac{\cos(\lambda^{*}L)\sinh(\lambda^{*}L)+\sin(\lambda^{*}L)\cosh(\lambda^{*}L)}{2\left(1+\cos(\lambda^{*}L)\cosh(\lambda^{*}L)\right)}$$

These parameters are frequency-dependent because the eigenvalue $\lambda^{*}$ is too. From Equation (11), the transmissibility function was obtained and used to validate results.

## 4. Analysis of the Four Curve-Fitting Methods

To validate and to analyse the results obtained by the curve-fitting method, the regenerated transmissibility function of the measuring point was obtained by means of (11) for x=L−5 mm. As the frequency-dependent complex modulus $E^{*}\left(f\right)$ was only measured at the resonance frequencies, a linear interpolation was used to obtain the regenerated signal in the whole frequency range. Four curve fitting cases were analysed:

- Case 1: multimodal without residuals, meaning that the terms of the fractions corresponding to $K^{*}$ and $M^{*}$ are 0 in Equation (2).
- Case 2: multimodal with residuals.
- Case 3: monomodal without residuals, meaning that the terms of the fractions corresponding to $K^{*}$ and $M^{*}$ are 0 in Equation (2).
- Case 4: monomodal with residuals.

**Figure 6:**
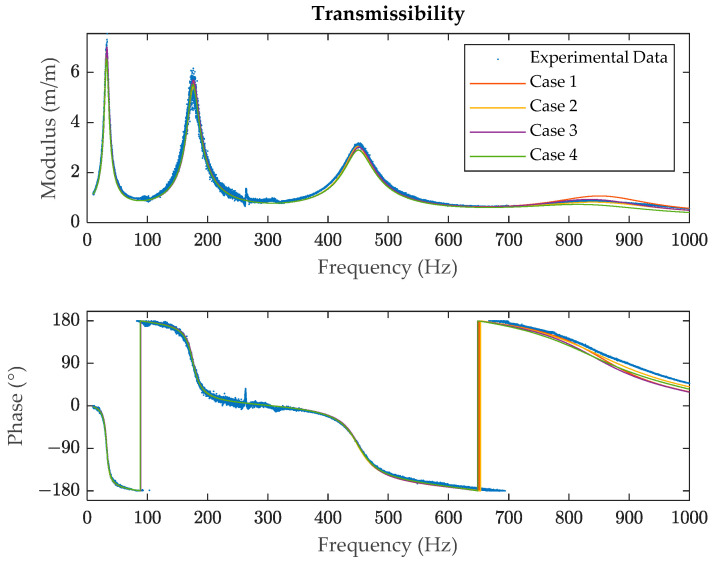
Experimental and regenerated transmissibility signals (modulus and phase) for the specimen from the curve-fitting method.

**Figure 7:**
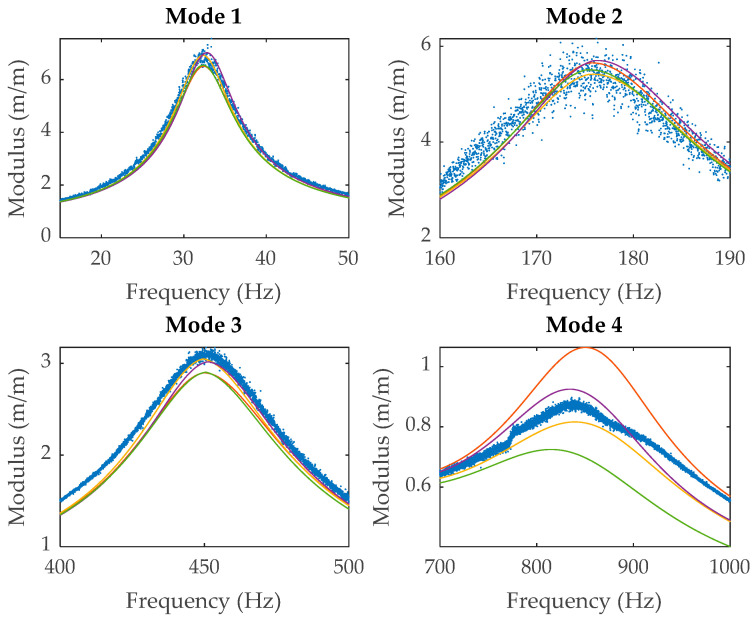
Transmissibility modulus detail for each resonance peak of the data shown in Figure 6 for: experimental data (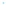), case 1 (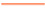), case 2 (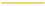), case 3 (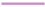) and case 4 (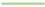).

The regenerated signals for the polymeric specimen are compared with the experimental results in Figure 6 and the numerical values for the resonance frequencies, storage moduli and modal loss factors of each resonance in Table 2 and Table 3. The transmissibility modulus for each resonance is shown in greater detail in Figure 7. The normalised difference between the experimental data and the regenerated function is indicated in Table 4 and given by

(16) $$e(x)=\frac{\sum_{k=1}^{k_{\max}}\left|T_{\exp,k}^{*}-T^{*}\left(f_{k},x\right)\right|}{k_{\max}}$$

From the results, it can be seen that the modal loss factor is the most affected parameter for each case and that the first and the fourth resonances are the most sensitive ones. It can be pointed out too that there are barely any significant differences between the regenerated signals for each of the four cases, except for the last resonance. It can also be stablished that case 2 is the best one at regenerating the original signal and case 3 the worst one according to the differences shown in Table 4.

According to the results observed in Figure 6, case 2 provides the best results at the fourth resonance, whereas case 1 underestimates the damping and case 4 overestimates it, the differences between the modal loss factor for case 1 and case 4 being 44%. Thus, it can be concluded that when the damping of polymeric materials is high, residuals cannot be ignored and that considering all resonances simultaneously is preferred. Although some minor differences are also observed for the second resonance between each case shown in Table 2 and Table 3, this can be explained by the noise that the experimental data have. The variation of the results for the frequencies, storage moduli and modal loss factors is less than 10% in all other cases. 

Concluding, the study performed allowed us to establish a general robust methodology when performing experimental tests to characterise viscoelastic materials based on the curve-fitting method. This method required minimising Equation (3) with an iterative method where a guess value had to be provided for each unknown parameter. For this reason, it was proposed to use the curve-fitting method as an SDOF method without residuals for each resonance first, to obtain an initial approximation of the guess value. That initial guess value was then used when applying the curve-fitting method to the whole frequency range by means of the MDOF method with residuals.

## 5. Conclusions

In this paper, a polymeric beam was tested to obtain the frequency-dependent complex modulus of a polymeric material. The experimental setup was presented, and an in-depth study was performed on the curve-fitting method used, where four cases were analysed.

Results were validated by regenerating the original signal from the analytical expression given by the Euler–Bernoulli beam theory. The modal loss factor was the most sensitive parameter according to the results obtained. Furthermore, as the damping of the polymeric material increased, the iterative curve-fitting method could regenerate the original signal accurately when considering an MDOF method with residuals.

For determining the guess or initial values we proposed to use the SDOF method without residuals on each resonance peak first, and then use the MDOF method. According to the results, it can be established that the proposed method presented great advantages compared to other traditional methods such as the HPB method for polymeric material testing where a high damping or noise levels can limit their use, whereas the curve-fitting method proved to be a solid choice. We conclude that the best way to apply the curve-fitting method for further testing of polymeric materials is when it is used on the whole frequency range by means of the MDOF method considering the residuals. In addition, this has the added advantage that the number of experimental tests to be carried out is much lower compared to using this method resonance by resonance.

## Figures and Tables

**Table 1:** Geometry and physical properties of the polymeric specimen.

$\begin{array}{c} L_{\mathrm{tot}}\\ (\pm 0.5\;\mathrm{mm}) \end{array}$	$\begin{array}{c} b\\ (\pm 0.05\;\mathrm{mm}) \end{array}$	$\begin{array}{c} H\\ (\pm 0.01\;\mathrm{mm}) \end{array}$	$\begin{array}{c} m\\ (\pm 0.05\;g) \end{array}$	$\begin{array}{c} L\\ (\pm 0.5\;\mathrm{mm}) \end{array}$
200.0	9.90	1.47	23.20	150.0

**Table 2:** Results of the curve-fitting method for the polymeric specimen for cases 1 and 2.

Resonance	Case 1			Case 2		
	$f_{s}\;(\mathrm{Hz})$	$E_{s}\;\left(\mathrm{GPa}\right)$	$\eta_{s}$	$f_{s}\;(\mathrm{Hz})$	$E_{s}\;\left(\mathrm{GPa}\right)$	$\eta_{s}$
1	32.08	73.55	0.233	32.04	73.38	0.219
2	175.3	55.91	0.129	175.3	55.93	0.135
3	449.9	46.98	0.129	449.6	46.93	0.123
4	855.5	44.24	0.210	857.1	44.40	0.270

**Table 3:** Results of the curve-fitting method for the polymeric specimen for cases 3 and 4.

Resonance	Case 3			Case 4		
	$f_{s}\;(\mathrm{Hz})$	$E_{s}\;\left(\mathrm{GPa}\right)$	$\eta_{s}$	$f_{s}\;(\mathrm{Hz})$	$E_{s}\;\left(\mathrm{GPa}\right)$	$\eta_{s}$
1	32.52	75.56	0.216	32.02	73.29	0.231
2	175.8	56.26	0.128	175.0	55.72	0.133
3	451.2	47.25	0.124	450.4	47.09	0.129
4	844.2	43.07	0.240	838.8	42.53	0.303

**Table 4:** Normalised difference between experimental data and regenerated signals for each analysed case.

Reference Case	e(x)
Case 1	0.1464
Case 2	0.1148
Case 3	0.1536
Case 4	0.1527

## References

[1] Fernandez-Garcia M., Muñoz-Bonilla A., Echeverria C. (2019). Polymeric Materials: Surfaces, Interfaces and Bioapplications. Materials.

[2] Ehrenstein G.W. (2001). Polymeric Materials.

[3] Brazel C.S., Rosen S.L. (2012). Fundamental Principles of Polymeric Materials.

[4] Lin S.Y. (2016). An Overview of Advanced Hyphenated Techniques for Simultaneous Analysis and Characterization of Polymeric Materials. Crit. Rev. Solid State Mater. Sci..

[5] Di Paola M., Galuppi L., Royer Carfagni G. (2021). Fractional Viscoelastic Characterization of Laminated Glass Beams under Time-Varying Loading. Int. J. Mech. Sci..

[6] Centelles X., Pelayo F., Lamela-Rey M.J., Fernández A.I., Salgado-Pizarro R., Castro J.R., Cabeza L.F. (2021). Viscoelastic Characterization of Seven Laminated Glass Interlayer Materials from Static Tests. Constr. Build. Mater..

[7] Fahey S.O.F., Pratt J. (1998). Time Domain Estimation Techniques. Exp. Tech..

[8] Fahey S.O.F., Pratt J. (1998). Frequency Domain Modal Estimation Techniques. Exp. Tech..

[9] Ewins D. (2000). Modal Testing: Theory, Practice and Application.

[10] Kadioglu F., Coskun T., Elfarra M. (2018). Investigation of Dynamic Properties of a Polymer Matrix Composite with Different Angles of Fiber Orientations. IOP Conf. Ser. Mater. Sci. Eng..

[11] Bulut M., Bozkurt Ö.Y., Erkliğ A., Yaykaşlı H., Özbek Ö. (2020). Mechanical and Dynamic Properties of Basalt Fiber-Reinforced Composites with Nanoclay Particles. Arab. J. Sci. Eng..

[12] Wesolowski M., Ruchwa M., Skukis E., Kovalovs A. (2020). Numerical and Experimental Extraction of Dynamic Parameters for Pyramidal Truss Core Sandwich Beams with Laminated Face Sheets. Materials.

[13] (1982). Brüel & Kjaer Sound and Vibration Measurement A/S Measurement of the Complex Modulus of Elasticity: A Brief Survey. Brüel Kjær Appl. Notes.

[14] (2005). Standard Test Method for Measuring Vibration-Damping Properties of Materials.

[15] Wu B. (2015). A Correction of the Half-Power Bandwidth Method for Estimating Damping. Arch. Appl. Mech..

[16] Wang J.T., Jin F., Zhang C.H. (2012). Estimation Error of the Half-Power Bandwidth Method in Identifying Damping for Multi-DOF Systems. Soil Dyn. Earthq. Eng..

[17] Davis S.P., Abrams M.C., Brault J.W. (2001). Fourier Transform Spectrometry.

[18] Brincker R., Ventura C.E. (2015). Introduction to Operational Modal Analysis.

[19] Avitabile P. (2006). Partv 5: 101 Ways to Extract Modal Parameters—Which One Is for Me?. Exp. Tech..

[20] Zrayka A.K., Mucchi E. (2019). A Comparison among Modal Parameter Extraction Methods. SN Appl. Sci..

[21] Martinez-Agirre M., Elejabarrieta M.J. (2011). Dynamic Characterization of High Damping Viscoelastic Materials from Vibration Test Data. J. Sound Vib..

[22] Xi Y., Wenhua Z., Yilin P., Wanting Z., Fenghao Y. (2021). Comparative Study on Damping Test Methods of Concrete Materials. Constr. Build. Mater..

[23] Inman D.J. (1996). Engineering Vibration.

[24] Cortés F., Elejabarrieta M.J. (2007). Viscoelastic Materials Characterisation Using the Seismic Response. Mater. Des..

